# Coverage evaluation of universal bacterial primers using the metagenomic datasets

**DOI:** 10.1186/1471-2180-12-66

**Published:** 2012-05-03

**Authors:** Dan-Ping Mao, Quan Zhou, Chong-Yu Chen, Zhe-Xue Quan

**Affiliations:** 1 Department of Microbiology and Microbial Engineering, School of Life Sciences, Fudan University, Shanghai, 200433, China

## Abstract

**Background:**

The coverage of universal primers for the bacterial 16S rRNA gene plays a crucial role in the correct understanding of microbial community structure. However, existing studies on primer coverage are limited by the lack of appropriate databases and are restricted to the domain level. Additionally, most studies do not account for the positional effect of single primer-template mismatches. In this study, we used 7 metagenomic datasets as well as the Ribosomal Database Project (RDP) to assess the coverage of 8 widely used bacterial primers.

**Results:**

The coverage rates for bacterial primers were found to be overestimated by previous studies that only investigated the RDP because of PCR amplification bias in the sequence composition of the dataset. In the RDP, the non-coverage rates for all primers except 27F were ≪6%, while in the metagenomic datasets, most were ≫10%. If one considers that a single mismatch near the 3′ end of the primer might greatly reduce PCR efficiency, then some phylum non-coverage rates would change by more than 20%. Primer binding-site sequence variants that could not pair with their corresponding primers are discussed.

**Conclusions:**

Our study revealed the potential bias introduced by the use of universal bacterial primers in the assessment of microbial communities. With the development of high-throughput, next-generation sequencing techniques, it will become feasible to sequence more of the hypervariable regions of the bacterial 16S rRNA gene. This, in turn, will lead to the more frequent use of the primers discussed here.

## Background

In the field of microbial ecology, the polymerase chain reaction (PCR) has been widely used for the amplification, detection and quantification of DNA targets since its introduction [1,2], resulting in increased knowledge of the microbial world [3,4]. However, the efficiency and accuracy of PCR can be diminished by many factors including primer-template mismatches, reactant concentrations, the number of PCR cycles, annealing temperature, the complexity of the DNA template, and others. [5-7]. Primer-template mismatches are the most important because they can lead to selective amplification which prevents the correct assessment of microbial diversity [8,9]. Target sequences that cannot match the primers precisely will be amplified to a lesser extent, possibly even below the detection limit. The relative content of the sequences achieved is therefore changed, resulting in a deviation from the true community composition. Hence a comprehensive evaluation of bacterial primer coverage is critical to the interpretation of PCR results in microbial ecology research.

Many related studies on primer coverage have been performed previously, but most are qualitative or semi-quantitative studies restricted to the domain level [10,11]. Low coverage rates in some rare phyla might have been overlooked.

Although Wang et al. [12] investigated primer coverage rates at the phylum level, only sequences from the Ribosomal Database Project (RDP) were used. This sole reliance on the RDP is another common limitation of previous studies. The RDP is a professional database containing more than one million 16S rRNA gene sequences. It also provides a series of data analysis services [13,14], including Probe Match, which is often used in primer studies. However, despite the RDP’s large collection of sequences and extensive application, most of its sequences were generated through PCR amplification. Sequences that fail to match the universal primers may become lost in the PCR results, and so are not included in the RDP. Consequently, primer coverage rates in the RDP appear to be higher than they actually are.

Fortunately, with the rapid development of sequencing techniques, many large-scale metagenomic datasets have become available. Metagenomic sequences are generated directly from sequencing environmental samples and are free of PCR bias; thus, the resulting datasets faithfully reflect microbial composition, especially in the case of rare biospheres. The Community Cyberinfrastructure for Advanced Microbial Ecology Research and Analysis (CAMERA) is not only a repository for rich and distinctive metagenomic data, but it also provides a set of bioinformatic tools for research[15].

Another shortcoming of previous primer-coverage studies has recently been illuminated through studies on the PCR mechanism. In the past, it was assumed that a single primer-template mismatch would not obstruct amplification under proper annealing temperature so long as the mismatch did not occur at the 3′ end of the primer. However, recent studies have shown that a single mismatch within the last 3–4 nucleotides of the 3′ end could also significantly reduce PCR amplification efficiency, even under optimal annealing temperature [16,17]. This changed the criteria for judging whether a primer binding-site sequence could be amplified faithfully by PCR. In this study, we define sequences that “match with” the primers as having either no mismatch with the primer, or as having only one mismatch that is not located within the last 4 nucleotides of the 3′ end.

All of the primers in this study are frequently used in molecular microbial ecology research. The most common primer pairs are 27F and 1390R/1492R, which are mainly used for constructing clone libraries of the full-length 16S rDNA sequence [18]. The primers such as 338F and 338R are frequently used in pyrosequencing [19-21]. The remaining primers are most commonly used for fingerprint analyses, but the development of next-generation sequencing techniques will likely broaden their roles in future studies [22,23]. Pyrosequencing has extended the read length from 100bp to 800bp [24], and as a result, hypervariable regions in 16S rDNA other than V6 and V3 will be able to be sequenced. Those primers that can cover these hypervariable regions will become more frequently used.

The aim of this study was to assess the coverage rates of 8 common primers (27F, 338F, 338R, 519F, 519R, 907R, 1390R and 1492R), which target different regions of the bacterial 16S rRNA gene, using sequences from the RDP and 7 metagenomic datasets. We used the non-coverage rate, the percentage of sequences that could not match with the primer, as the major indicator in this study. Non-coverage rates were calculated at both the domain and phylum levels, and the influence of a single mismatched position on the non-coverage rate was analyzed. By comparing the RDP and the metagenomic datasets, we found that the non-coverage rates were seriously underestimated when only the RDP dataset was used.

## Results and discussion

### Influence of a single mismatch in the last 4 nucleotides

Since the beginning of the 1990s, it has been widely acknowledged that PCR amplification is significantly inhibited by a single mismatch occurring at the 3′ end of the primer [25-27]. Even when the last nucleotide was substituted with inosine, which is capable of binding to all four nucleotides, primers still failed to amplify all of the expected sequences in the microbial community [28]. Recently, Bru et al. [16] and Wu et al. [17] demonstrated that the efficiency of PCR amplification was also inhibited if a single mismatch occurred within the last 3–4 nucleotides of the 3′ end of primer, even when the annealing temperature was decreased for optimal efficiency. These single mismatches have not been considered in previous primer coverage studies [12,18,29].

**Figure 1:**
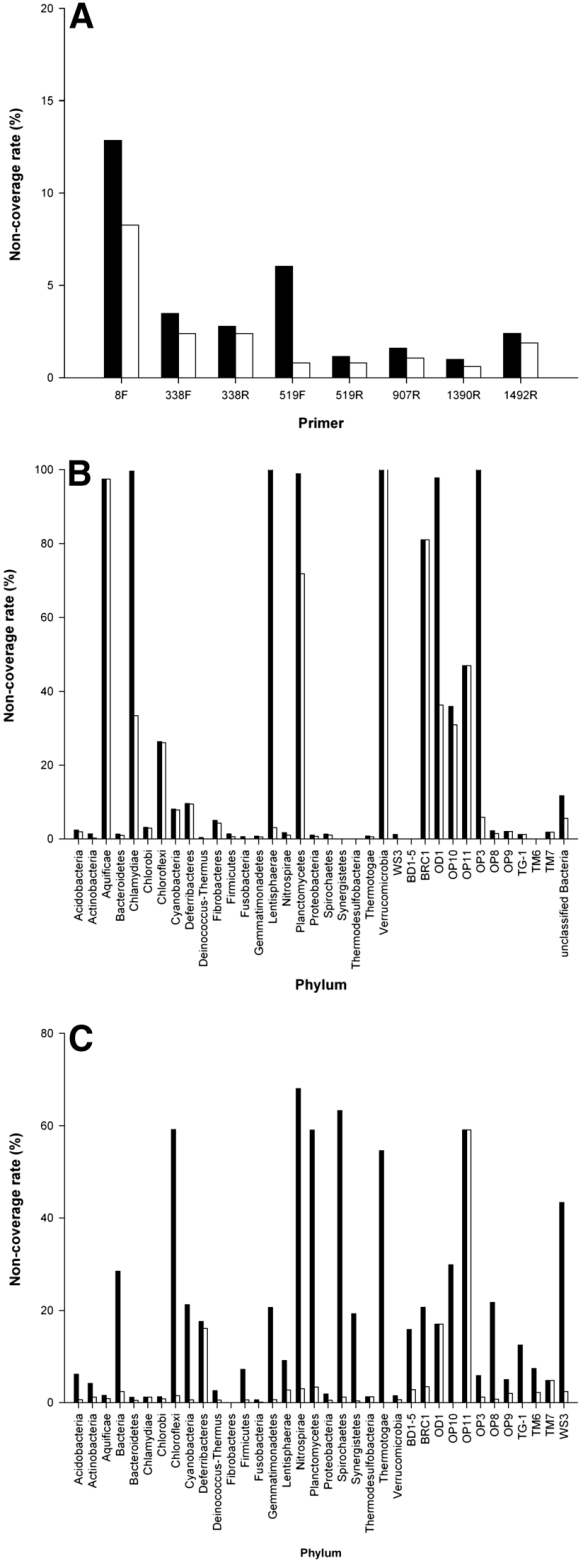
**Influence of a single mismatch occurring in the last 4 nucleotides.** The black column denotes the non-coverage rate when no mismatches were allowed in the last 4 nucleotides, while the white column denotes the rate when a single mismatch was allowed. **A** Domain non-coverage rates for 8 primers in the RDP dataset; **B** Phylum non-coverage rates for primer 338 F in the RDP dataset; **C** Phylum non-coverage rates for primer 519 F in the RDP dataset. Refer to Additional file 1: Figure S1A for the normalized results of Figure 1A.

We studied the influence of a single primer mismatch occurring within the last 4 nucleotides using the RDP dataset. At the domain level, a relatively weak influence was found when non-coverage rates that allowed a single mismatch in the last 4 nucleotides were compared to rates that did not allow such a mismatch. The absolute differences were ≪5% for all of the primers except 519F (Figure 1A). In contrast, significant differences were observed for some of the primers at the phylum level. Rate differences ≫20% under two criteria are listed in Table 1. The most noticeable non-coverage rate was observed for 338F in the phylum *Lentisphaerae*. If a single mismatch was allowed within the last 4 nucleotides, its non-coverage rate was only 3%; otherwise, it was as high as 100%. Similar results were observed for 338F in the phylum OP3, but with a smaller number of sequences. These results indicate that 338F is not appropriate for either phylum (*Lentisphaerae* or OP3). Overall, the most seriously affected primer was 519F. In this case, 10 phyla showed rate differences ≫20% under two criteria, and 6 phyla showed differences ≫40%. The significant differences observed at the phylum level imply that a single mismatch in the last 4 nucleotides may be fatal under specific circumstances, and this possibility should be considered when choosing and designing primers.

**Table 1:** Influence of a single mismatch near the 3′ end in the RDP dataset

**Primer**	**Phylum**	**Non-coverage rate 4+ (%)**	**Non-coverage rate 4- (%)**
338 F	*Lentisphaerae*	3.0	100.0
	OP3	5.9	100.0
	*Chlamydiae*	33.5	99.6
	OD1	36.3	97.8
	*Planctomycetes*	71.9	98.9
519 F	*Nitrospirae*	3.0	68.1
	*Spirochaetes*	1.2	63.3
	*Chloroflexi*	1.5	59.2
	*Planctomycetes*	3.4	59.1
	*Thermotogae*	0.0	54.6
	WS3	2.4	43.4
	OP10	0.0	29.8
	OP8	0.7	21.7
	*Cyanobacteria*	0.6	21.3
	*Gemmatimonadetes*	0.6	20.7
	Unclassified Bacteria	2.4	28.4

At the phylum level, non-coverage rates that changed more than 20% under two criteria are listed. “Non-coverage rate 4+” denotes the non-coverage rate when a single mismatch in the last 4 nucleotides was allowed. “Non-coverage rate 4-” denotes the non-coverage rate when mismatches in the last 4 nucleotides were not allowed.

### Non-coverage rates of 8 primers at the domain level

**Figure 2:**
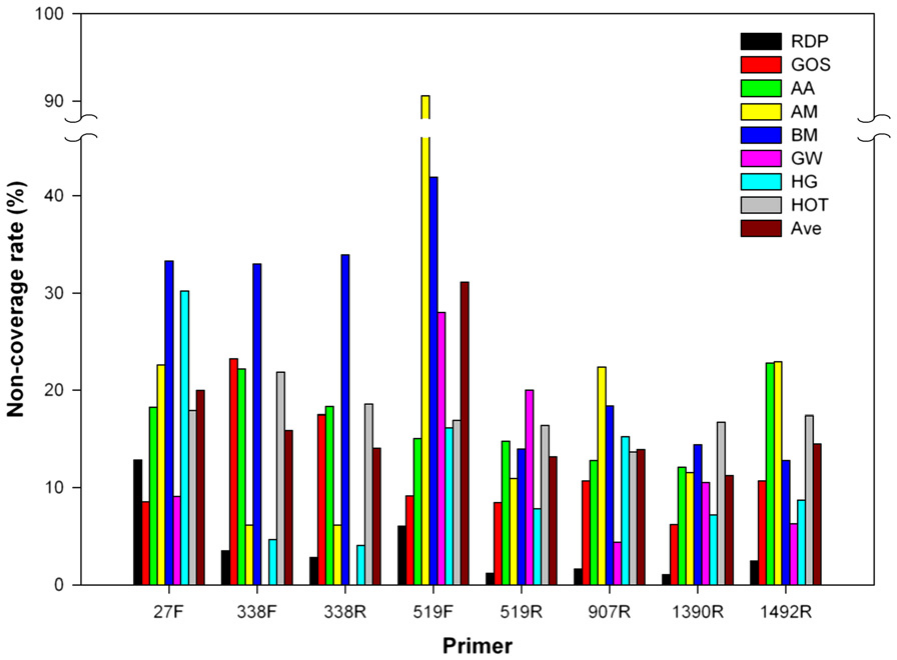
**Non-coverage rates at the domain level.** “AA” denotes the AntarcticaAquatic dataset, “AM” denotes the AcidMine dataset, “BM” denotes the BisonMetagenome dataset, “GW” denotes the GutlessWorm dataset, “HG” denotes the HumanGut dataset and “Ave” is the arithmetic mean of the 7 non-coverage rates of the metagenomic datasets. Mismatches in the last 4 nucleotides were not allowed. Refer to Additional file 1: Figure S1B for the normalized results. Refer to Additional file 2: Figure S2 for the phylum non-coverage rates.

Non-coverage rates for the 8 common primers relative to the 8 datasets examined were calculated (Figure 2). In the RDP dataset, the non-coverage rate for primer 27F reached 12.9%, but the rates of the other 7 primers were all ≪6%. However, in the metagenomic datasets, 40 out of 56 (8 primers multiplied by 7 metagenomic datasets) non-coverage rates were ≫10%. Moreover, for all primers except 27F, the average rates from the 7 metagenomic datasets were at least 4-times higher than in the RDP dataset, and the ratio even reached 11.4 for the primer 519R. Normalized results were similar (Additional file 1: Figure S1B). The average difference between the RDP and the metagenomic datasets was 12.82% before and 12.76% after normalization. The average absolute difference between the original and normalized domain non-coverage rates was 2.53%. These results revealed that the non-coverage rates in the RDP were greatly underestimated and proved the effectiveness of using metagenomes to assess primer coverage. Furthermore, after eliminating primer contamination (see Methods), most of the sequences containing a 27F binding site in the RDP came from the metagenomes. This might explain why the non-coverage rate for 27F in the RDP dataset was close to that in the metagenomic datasets.

### Non-coverage rates for 8 primers at the phylum level

Because each dataset is a mixture of sequences from various microbes occurring in various proportions according to different phyla, low coverage of minor phyla could be easily masked by the higher coverage of the dominant phyla. Moreover, the compositions of microbial communities differ greatly with environments; Minor microbes found in common environments may in fact be major components in other ecological niches. It is therefore necessary to assess the non-coverage rates at the phylum level in the different metagenomic datasets.

#### *338F and 338R*

Non-coverage rates for the primers 338F and 338R varied among different phyla (Additional file 2: Figure S2.). In the RDP dataset, the non-coverage rates for 338F in 4 phyla (*Aquificae*, *Planctomycetes*, *Verrucomicrobia* and OD1) were ≫95%. Primer binding-site sequences that could not match with primer 338F are listed in Additional file 3: Table S2.

In the RDP dataset, the most frequent sequence variant retrieved (3,587 sequences) was 338F-3A12T (3A indicates that the 3rd base is the nucleotide A, and 12T that the 12th base is the nucleotide T). This sequence was the major variant in the *Verrucomicrobia*, accounting for 97.8% of the sequences in the RDP dataset and 85.7% in the GOS (Global Ocean Sampling Expedition) dataset; it also predominated in the phyla *Chloroflexi*, BRC1, OP10 and OP11. The second variant, 338F-16T, was the major variant in the *Lentisphaerae* but also appeared in many other phyla. The third variant, 338F-3A12T16T, was specific for *Planctomycetes* and OD1, and accounted for approximately 50% of *Planctomycetes* in both the RDP and GOS datasets. The variants 338F-4T11A and 338F-12G were distributed in various phyla, while 338F-3C12G was specific for *Aquificae* and 338F-3C4T11A12G for *Cyanobacteria*.

Also significant was the non-coverage rate for 338F in the *Actinobacteria*. In the RDP dataset, this rate was only 1.3%, but in the metagenomic datasets, the results were substantially different. The non-coverage rates in the GOS and HOT datasets, for example, were 60.4% and 66.7%, respectively. We observed that the absolute number of 338F-16T sequences from *Actinobacteria* in the RDP dataset was 631, which was much larger than the numbers in the GOS and HOT datasets. The implication is that the 338F-16T *Actinobacteria* sequences in the RDP most likely came from environments similar to those from which the GOS and HOT sequences were sampled.

For the primer 338R, the reverse complement of 338F, the homologous variants 338F-16T and 338F-16C had no effect on the non-coverage rate, while three other variants (338R-16G, 338R-18C and 338R-15A) warranted further attention (Additional file 3: Table S3). Although hundreds of sequences for each variant were found, they accounted for low percentages of the major phyla (*Actinobacteria*, *Bacteroidetes*, *Firmicutes* and *Proteobacteria*). Variants with more than one mismatch were similar to those of 338F.

The BisonMetagenome dataset was dominated by *Aquificae* and the non-coverage rates for both 338F and 338R in *Aquificae* were 100%. The sequence variant 338F-3C12G (338R-7C16G) was the major type. Thus, the primers 338F/338R might not be appropriate for the analysis of hot spring samples or the detection of *Aquificae.*

#### *519F and 519R*

The coverage of primer 519R was quite “universal” except for its high non-coverage rate in the phylum OD1 in the AntarcticaAquatic dataset, where the primer binding-site sequence variant 519R-14T-11T12C had a rate of 84.6%. Although non-coverage rates of approximately 20% were found scattered across other phyla, these rates resulted from variants with only one or two sequences, and no dominating variant was found. Overall, primer 519R could authentically amplify sequences from most phyla.

A substantial difference was found between the non-coverage rates of 519F and 519R. Five sequence variants were mainly responsible for the high non-coverage rate for 519F (Additional file 3: Table S4). Notably, the 3 most dominant variants had one trait in common – a single mismatch at the 16th nucleotide (the 3rd nucleotide from the 3′ end of 519F). This mismatch did not influence the non-coverage rate of 519R.

Further analysis showed that the high non-coverage rate of 519F was caused primarily by sequences from the phylum *Nitrospirae*. The AcidMine metagenome is dominated by *Leptospirillum* species of th*e Nitrospirae,* and therefore forms an ideal dataset for *Nitrospirae* studies [30]. Of the 519F-binding sequences in the dataset, 89% were from *Nitrospirae*, and none could match with 519F. The non-coverage rate in the RDP dataset was also high (68%) in *Nitrospirae*, whereas the total non-coverage rate for 519F in the RDP dataset was only 6%. Similar sample analyses should therefore be focused on the use of primer 519F.

#### *Other primers*

Frank et al. [18] have studied the 27F and 1492R primer pair and have proposed 27F-YM + 3 as a modification of the common 27F primer. Our results support this modification as being necessary (Additional file 3: Table S1). The non-coverage rates for 1390R and 1492R were quite low, even at the phylum level. For primer 907R, only one sequence variant that could not match with the primer (907R-11C-15A16T) was observed. It resulted in the high non-coverage rate observed in phylum TM7 (Additional file 3: Table S5).

## Conclusions

The 16S rRNA gene is an important genetic marker for the characterization of microbial community structure by 16S rRNA gene amplicon sequencing with conserved primers [31]. Because of the increase in read length with the development of pyrosequencing (454 sequencing) technology, different multi-hypervariable regions can be selected for amplification. In this strategy, different pairs of “universal” primers are used for barcoded pyrosequencing [32]. However, even with pyrosequencing, the bias caused by primer-template mismatch may misrepresent the real community composition of environmental samples. Therefore, the assessment of primer coverage to perfect the use of universal primers is urgently required.

In this study, we assessed the non-coverage rates for 8 common universal bacterial primers in the RDP dataset and 7 metagenomic datasets. Comparisons of non-coverage rates, with or without constraining the position of a single mismatch, emphasized the importance of further study of the mechanism of PCR. Metagenomic dataset analysis revealed that some sequence variants, which appeared to be minor in the public databases, were actually dominant in some ecological niches. These results are of great practical significance for studies on similar environmental samples, and new primer formulations could be designed using our results. One strategy is to increase coverage through the introduction of proper degenerate nucleotides.

Although the total number of sequences in a metagenomic dataset may be very large, the number of 16S rRNA gene sequences is limited, and may account for only approximately 0.2% of all sequence reads [33,34]. In contrast, the metatranscriptomic analysis of environmental samples generates a large number of small subunit sequences [35]. Although the short length (approximately 200bp) of the sequences currently deposited in metatranscriptomic datasets are not appropriate for assessing primer coverage, the further development of pyrosequencing will make such assessments possible in the near future.

## Methods

### Retrieval of 16S rRNA gene sequences from the RDP

A FASTA file for all bacterial 16S rRNA gene sequences was downloaded from the “RESOURCES” section of the RDP website (release 10.18; http://rdp.cme.msu.edu/) [14]. With the help of the service “BROWSERS”, good quality, almost full-length (size ≥ 1200bp) sequences were obtained. These sequences were extracted from the FASTA file by Perl scripts. A final dataset with 462,719 bacterial 16S rRNA gene sequences was constructed (referred to as the “RDP dataset”).

### Elimination of primer contamination in the RDP dataset

Most sequences deposited in the RDP dataset were generated by PCR. However, as described by Frank et al. [18], many of these sequences lack correct primer trimming. Only sequence fragments extending at least 3 nucleotides past the start (the 5′ end) of the longest version of each primer were considered uncontaminated by the PCR primers. Because the sequences selected from the RDP were all longer than 1200bp, only the primer-binding sites for 27F, 1390R and 1492R could be contaminated (Additional file 4: Figure S3). Thus, 15,045, 188,792 and 35,462 sequences were selected for the primers 27F, 1390R and 1492R, respectively, as containing authentic primer-binding sites.

### Retrieval of 16S rDNA sequences from the metagenomic datasets

#### *Selection of metagenomic datasets*

Metagenomic datasets were selected from the CAMERA website (release v.1.3.2.30; http://camera.calit2.net/) [15]. Given the read length and the diversity of sample sources, 7 microbial metagenomic datasets constructed by shotgun sequencing were chosen (average sequence length ≫ 900bp, sequence number ≫ 300,000): AntarcticaAquatic, AcidMine, BisonMetagenome, GOS, GutlessWorm, HumanGut and HOT. Detailed descriptions for each dataset are listed in Table 2.

**Table 2:** Descriptions of the metagenomic datasets

**Project name**	**Source description**	**Dominating phylum/phyla**	**Reference**
AntarcticaAquatic (AA)	Antarctica Aquatic Microbial Metagenome (All Metagenomic Shotgun Reads)	*Bacteroidetes, Proteobacteria*	[36]
AcidMine (AM)	Acid Mine Drainage Metagenome (All Metagenomic Shotgun Reads)	*Nitrospirae*	[30]
BisonMetagenome (BM)	Metagenome from Yellowstone Bison Hot Spring (All Metagenomic Shotgun Reads)	*Aquificae*	[37]
GOS	Global Ocean Sampling Expedition (All Metagenomic Sequence Reads)	*Proteobacteria*	[38]
GutlessWorm (GW)	Mediterranean Gutless Worm Metagenome (All Metagenomic Sequence Reads)	*Proteobacteria*	[39]
HumanGut (HG)	Human Distal Gut Biome project (Assembled Sequences)	*Firmicutes, Actinobacteria*	[40]
HOT	Microbial Community Genomics at the Hawaii Ocean Time-series (HOT) station ALOHA (All Metagenomic Sequence Reads)	*Proteobacteria, Cyanobacteria*	[41]

The metagenomic datasets used in this paper are from the CAMERA website (http://camera.calit2.net/). Dominating phyla have sequences amounting to more than 20% of the total in the dataset.

#### *Retrieval of 16S rDNA homologs*

The Basic Local Alignment Search Tool (BLAST) was used to acquire as many 16S rRNA gene homologs as possible for the low content of such sequences in the metagenomic datasets. A query set of 34 representative and almost full-length 16S rRNA gene sequences from 34 bacterial phyla was constructed. BLAST searches using the query set and each selected dataset were performed using the CAMERA interface (db alignments per query, 50000; e-value exponent (1Ex), -5; filter low-complexity seq, T; lower case filtering, False). For the GOS dataset, BLAST was performed using each query sequence separately because the subjects exceeded the threshold of “db alignments per query” when BLAST was performed using the complete query set. After removing reads containing the nucleotide “N”, sequence reads were merged into one file without duplication. Seven files were obtained, one from each of the 7 datasets.

#### *Further filtration of 16S rDNA homologs*

The software program Mothur (http://www.mothur.org) was used for further filtration [42]. Sequences and their reverse complements were aligned separately via the command “align.seqs”. One reference file containing large subunit rRNA gene sequences was downloaded from Silva (http://www.arb-silva.de/) [43]. The second reference file was a combination of Silva reference files of small subunit rRNA gene sequences downloaded from Mothur. According to the alignment scores, the origin and direction of the sequences were ascertained. Sequences whose scores were always ≪30 might represent non-rRNA genes and were therefore removed.

For the RDP dataset, the alignment with the reference file of small subunit rDNA sequences was run first, and sequences with alignment scores ≪30 were removed.

### Taxonomic assignment

The 16S rRNA gene sequences from both the RDP dataset and the metagenomic datasets were assigned to different taxonomic groups by Mothur, with the confidence threshold set at 80%. Sequences classified as belonging to the domain Bacteria were listed and extracted.

### Identification of primer-binding sites in 16S rDNA sequences

Because the alignment using the Silva template sequences did not include the entire length of the gene, thus missing the primer-binding sites for 27F and 1492R, alignment with another reference file (the “Core Set” of the Greengenes database) was used to identify the primer-binding sites [44]. A full-length 16S rRNA gene sequence from *Escherichia coli* (GenBank ID: J01695) was added for base positioning.

Eight primers were selected (see Table 3 for detailed information) and primer-binding sites were extracted by Perl script. To avoid the base slip caused by multiple sequence alignment, the extraction was not precise, but was made with 5 additional bases at both ends. Primer-binding site sequences that were incomplete, or which contained ambiguous nucleotides, were discarded. Comparisons between the primer-binding site and its corresponding primer were performed using Probe Match (ARB) [45].

**Table 3:** Detailed information for the 8 primers evaluated

**Primer name**	**Degenerate type**	**Sequence of primer**	**Position in *Escherichia coli***	**Reference (s)**
27 F (8 F)	11Y12M	5′- AGA GTT TGA T**YM** TGG CTC AG-3′	8-27	[46]
338 F		5′-ACT CCT ACG GGA GGC AGC-3′	338-355	[47]
338R		5′-GCT GCC TCC CGT AGG AGT-3′	355-338	[48]
519 F	5 M	5′-CAG C**M**G CCG CGG TAA TAC-3′	519-536	[49]
519R (536R)	14 K	5′-GTA TTA CCG CGG C**K**G CTG-3′	536-519	[50]
907R (926R)	11 M	5′-CCG TCA ATT C**M**T TTG AGT TT-3′	926-907	[51]
1390R (1406R)	14R	5′-ACG GGC GGT GTG T**R**C AA-3′	1390-1406	[1,52]
1492R	11Y	5′-TAC CTT GTT A**Y**G ACT T-3′	1492-1507	[53,54]

Alternative names for the primers are annotated in parentheses. In the “Degenerate type” column, the number and the capital letter denote the position and the content of the degenerate nucleotides. For example, primer 27 F is also known as 8 F, and “11Y12M” means that the 11th base is the degenerate nucleotide Y and the 12th base is M (Y = C or T, M = A or C, K = T or G and R = A or G).

### Data analysis

Primer binding-site sequences with more than one mismatch, or with a single mismatch within the last 4 nucleotides of the 3′ end, were considered unmatched with the primer. Non-coverage rates were calculated as the percentage of such sequences. The non-coverage rates of phyla with sequence numbers of less than 50 in the RDP dataset or less than 10 in the metagenomic datasets were not shown in Figure 1 and Additional file 2: Figure S2.

Because different phyla vary considerably in the numbers of sequences reported, we attempted a normalization approach to calculate the non-coverage rates for each dataset. Phyla with less than 10 sequences or 1% of the total of each dataset were merged into a new “phylum”. The domain non-coverage rate was computed as the arithmetical average of the phylum non-coverage rates.

## Authors’ contributions

DPM, QZ and ZXQ conceived of, designed and performed the experiments. DPM, QZ, CYC and ZXQ analyzed the data. DPM, QZ and ZXQ wrote the paper. All authors read and approved the final manuscript.

## Supplementary Material

Additional file 1 **Figure S1. Normalized non-coverage rates.** A Normalized domain non-coverage rates in the RDP dataset for Figure 1A; B Normalized domain non-coverage rates for Figure 2. Click here for file

Additional file 2 **Figure S2. Non-coverage rates at the phylum level.** The figures show the non-coverage rates of different primers at the phylum level: A Primer 27F; B Primer 338F; C Primer 338R; D Primer 519F; E Primer 519R; F Primer 907R; G Primer 1390R; and H Primer 1492R. Click here for file

Additional file 3 **Table S1; Table S2; Table S3; Table S4; Table S5. Primer binding-site sequence variants.** Frequently observed sequence variants at different primer binding sites are listed in different tables: **Table S1** Primer 27F; **Table S2** Primer 338F; **Table S3** Primer 338R; **Table S4** Primer 519F; **and Table S5** Primer 907R. Click here for file

Additional file 4 **Figure S3. Elimination of primer contamination.** The figure shows the elimination of sequences that are thought to lack correct primer trimming in the RDP dataset. Click here for file
