# Supplementary material for: Coverage evaluation of universal bacterial primers using the metagenomic datasets
Source: BMC Microbiol. 2012 May 3;12:66. doi: 10.1186/1471-2180-12-66 (PMC3445835; doi:10.1186/1471-2180-12-66)
Supplement: Additional file 1 — Figure S1. Normalized non-coverage rates. A Normalized domain non-coverage rates in the RDP dataset for Figure 1A; B Normalized domain non-coverage rates for Figure 2. [file 1471-2180-12-66-S1.doc]

**
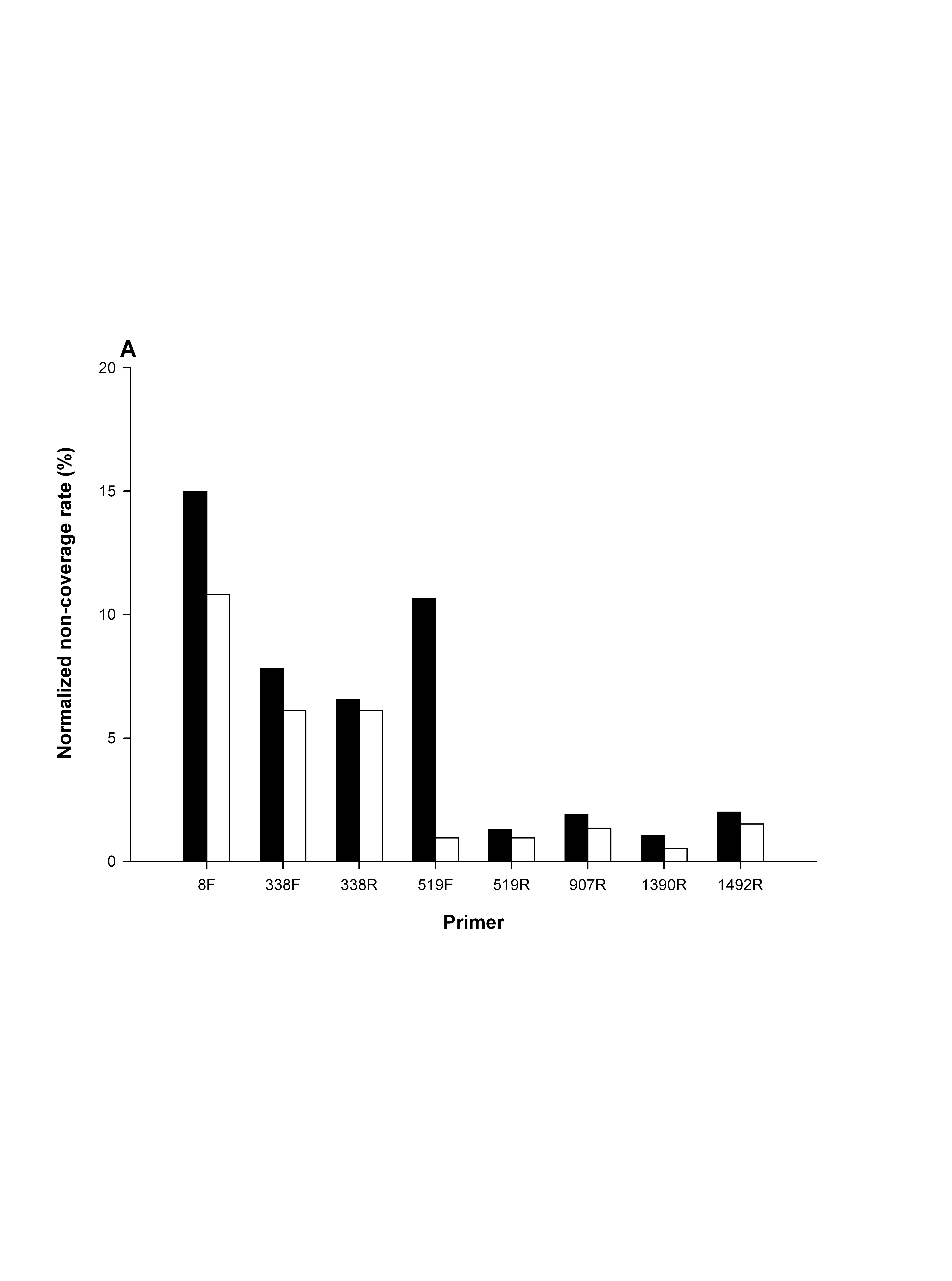
**

**
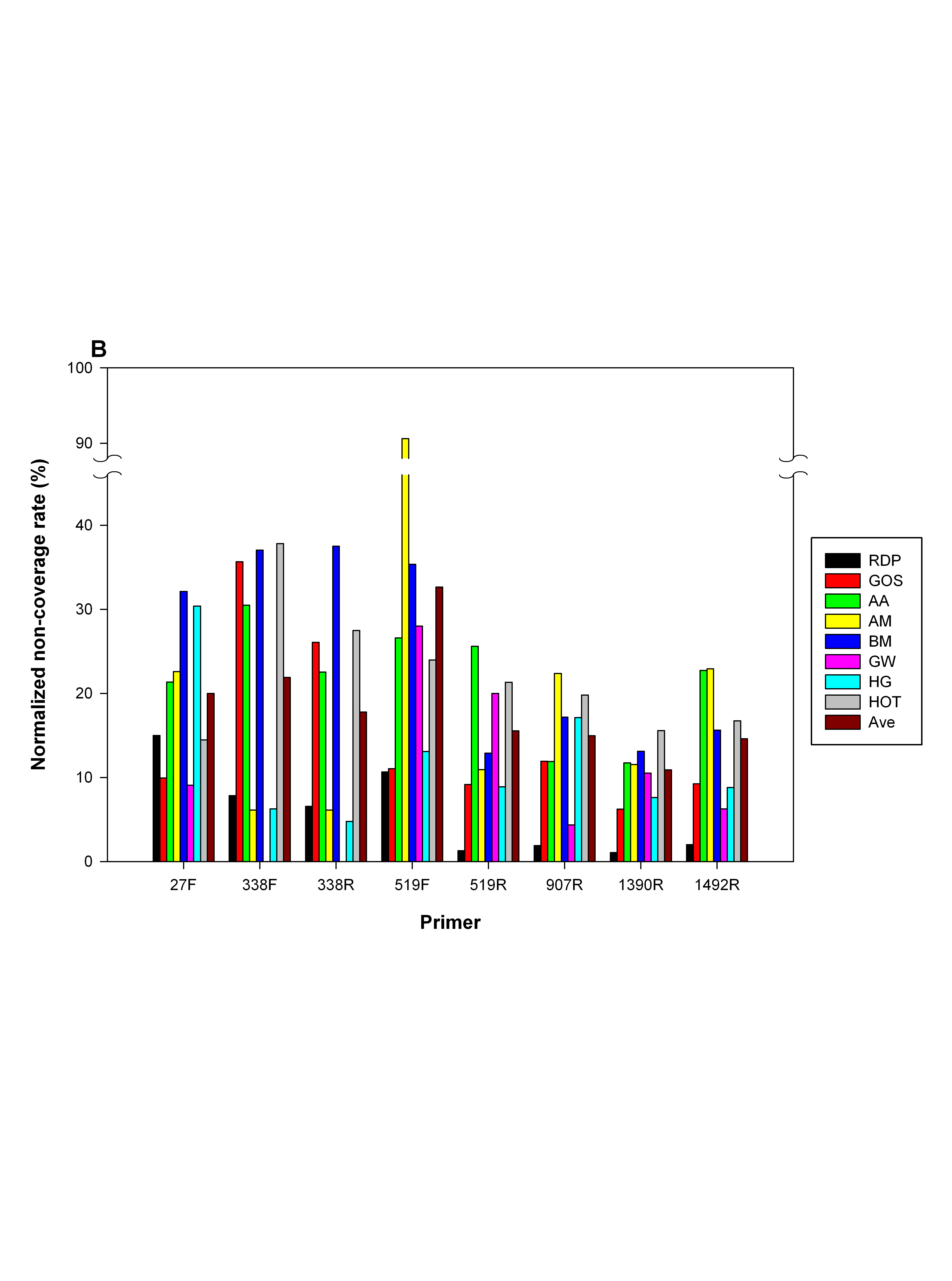
**

**Figure S1. Normalized non-coverage rates**

**A** Normalized domain non-coverage rates for Figure 1A. The figure shows the influence of a single mismatch in the last 4 nucleotides in the RDP dataset. The black column denotes the non-coverage rate when no mismatches in the last 4 nucleotides were allowed, while the white column denotes the rate when a single mismatch was allowed.

**B** Normalized domain non-coverage rates for Figure 2.

“AA” denotes the AntarcticaAquatic dataset, “AM” denotes the AcidMine dataset, “BM” denotes the BisonMetagenome dataset, “GW” denotes the GutlessWorm dataset, “HG” denotes the HumanGut dataset, and “Ave” is the arithmetic mean of 7 non-coverage rates of the metagenomic datasets. Mismatches within the last 4 nucleotides were not allowed.
