# Supplementary material for: Coverage evaluation of universal bacterial primers using the metagenomic datasets
Source: BMC Microbiol. 2012 May 3;12:66. doi: 10.1186/1471-2180-12-66 (PMC3445835; doi:10.1186/1471-2180-12-66)
Supplement: Additional file 2 — Figure S2. Non-coverage rates at the phylum level. The figures show the non-coverage rates of different primers at the phylum level: A Primer 27F; B Primer 338F; C Primer 338R; D Primer 519F; E Primer 519R; F Primer 907R; G Primer 1390R; and H Primer 1492R. [file 1471-2180-12-66-S2.doc]

**Table S1.** **Major sequence variants at the 27F binding site.** In the “Name of the sequence variant” column, the number and capital letter after the first hyphen denote the position and nucleotide that are accordant with the degeneracy of the standard primer, while the number and capital letter following the second hyphen denote the position and nucleotide that are different from the standard primer. In the second column, nucleotides different from the standard primer appear in bold.

| Name of the sequence variant | Primer sequence  5’-AGA GTT TGA T**YM** TGG CTC AG-3’ | Dataset | Phylum | Number of the sequence variant | Number of sequences in the phylum | Percentage of the sequence variant in the phylum |
| --- | --- | --- | --- | --- | --- | --- |
| 27F-11C12C-20A | AGA GTT TGA TCC TGG CTC A**A** | RDP | *Bacteroidetes* | 45 | 990 | 4.5 |
|  |  |  | *Firmicutes* | 437 | 7805 | 5.6 |
|  |  |  | *Verrucomicrobia* | 7 | 34 | 20.6 |
| 27F-11C-4A12T16T | AGA **A**TT TGA TC**T** TGG **T**TC AG | RDP | *Chlamydiae* | 68 | 73 | 93.2 |
| 27F-11C12C-18T | AGA GTT TGA TCC TGG CT**T** AG | RDP | *Spirochaetes* | 18 | 44 | 40.9 |
| 27F-11T12C-3G7C | AG**G** GTT **C**GA TTC TGG CTC AG | RDP | *Actinobacteria* | 22 | 10000 | 0.2 |
|  |  | HumanGut | *Actinobacteria* | 41 | 56 | 73.2 |

**Table S2.** **Major sequence variants at the 338F binding site.** In the “Name of the sequence variant” column, the number and capital letter after the hyphen denote the position and nucleotide that are different from the standard primer. In the second column, nucleotides different from the standard primer appear in bold.

| Name of the sequence variant | Primer sequence  5’-ACT CCT ACG GGA GGC AGC-3’ | Dataset | Phylum | Number of the sequence variant | Number of sequences in the phylum | Percentage of the sequence variant in the phylum |
| --- | --- | --- | --- | --- | --- | --- |
| 338F-3A12T | AC**A** CCT ACG GG**T** GGC AGC | RDP | *Verrucomicrobia* | 2891 | 2955 | 97.8 |
|  |  |  | *Chloroflexi* | 537 | 2908 | 18.5 |
|  |  |  | BRC1 | 43 | 58 | 74.1 |
|  |  |  | OP10 | 53 | 181 | 29.3 |
|  |  |  | OP11 | 36 | 113 | 31.9 |
|  |  | GOS | *Verrucomicrobia* | 24 | 28 | 85.7 |
| 338F-16T | ACT CCT ACG GGA GGC **T**GC | RDP | *Actinobacteria* | 631 | 83391 | 0.8 |
|  |  |  | OD1 | 104 | 223 | 46.6 |
|  |  |  | OP1 | 20 | 28 | 71.4 |
|  |  |  | OP3 | 79 | 85 | 92.9 |
|  |  |  | *Planctomycetes* | 807 | 2999 | 26.9 |
|  |  |  | *Chlamydiae* | 164 | 248 | 66.1 |
|  |  |  | *Lentisphaerae* | 571 | 591 | 96.6 |
|  |  |  | *Firmicutes* | 552 | 133380 | 0.4 |
|  |  |  | Unclassified Bacteria | 181 | 3211 | 5.6 |
|  |  | GOS | *Actinobacteria* | 246 | 502 | 49.0 |
|  |  | HOT | *Actinobacteria* | 8 | 15 | 53.3 |
| 338F-3A12T16T | AC**A** CCT ACG GG**T** GGC **T**GC | RDP | *Planctomycetes* | 1533 | 2999 | 51.1 |
|  |  |  | OD1 | 45 | 223 | 20.2 |
|  |  | GOS | *Planctomycetes* | 5 | 10 | 50.0 |
|  |  |  | OD1 | 9 | 21 | 42.9 |
| 338F-4T11A | ACT **T**CT ACG G**A**A GGC AGC | RDP | *Proteobacteria* | 272 | 125299 | 0.2 |
|  |  |  | *Firmicutes* | 157 | 133380 | 0.1 |
|  |  |  | *Cyanobacteria* | 93 | 6870 | 1.4 |
|  |  |  | *Chloroflexi* | 54 | 2908 | 1.9 |
|  |  |  | OP11 | 11 | 113 | 9.7 |
|  |  |  | *Bacteroidetes* | 225 | 45423 | 0.5 |
|  |  |  | Unclassified Bacteria | 37 | 3211 | 1.2 |
|  |  | GOS | *Bacteroidetes* | 42 | 372 | 11.3 |
|  |  |  | *Deferribacteres* | 6 | 84 | 7.1 |
|  |  |  | *Proteobacteria* | 88 | 2756 | 3.2 |
| 338F-3C12G | AC**C** CCT ACG GG**G** GGC AGC | RDP | *Aquificae* | 663 | 700 | 94.7 |
|  |  | BisonMetagenome | *Aquificae* | 19 | 20 | 95.0 |
| 338F-16G | ACT CCT ACG GGA GGC **G**GC | RDP | *Actinobacteria* | 149 | 83391 | 0.2 |
|  |  |  | *Bacteroidetes* | 61 | 45423 | 0.1 |
|  |  |  | *Firmicutes* | 178 | 133380 | 0.1 |
|  |  |  | *Proteobacteria* | 136 | 125299 | 0.1 |
| 338F-3C4T11A12G | AC**C T**CT ACG G**AG** GGC AGC | RDP | *Cyanobacteria* | 393 | 6870 | 5.7 |
| 338F-1G2A3C4T | **GAC T**CT ACG GGA GGC AGC | RDP | *Actinobacteri*a | 61 | 83391 | 0.1 |
|  |  |  | *Firmicutes* | 42 | 133380 | 0.0 |
|  |  |  | *Proteobacteria* | 77 | 125299 | 0.1 |
|  |  | GOS | *Bacteroidetes* | 17 | 372 | 4.6 |
|  |  |  | *Cyanobacteri*a | 29 | 394 | 7.4 |
| 338F-16C | ACT CCT ACG GGA GGC **C**GC | RDP | OD1 | 33 | 223 | 14.8 |
|  |  | AntarcticAquatic | OD1 | 5 | 10 | 50.0 |

**Table S3. Major sequence variants at the 338R binding site.** In the “Name of the sequence variant” column, the number and capital letter after the hyphen denote the position and nucleotide that are different from the standard primer. In the second column, nucleotides different from the standard primer appear in bold.

| Name of the sequence variant | Primer sequence  5’-GCT GCC TCC CGT AGG AGT-3’ | Dataset | Phylum | Number of the sequence variant | Number of sequences in the phylum | Percentage of the sequence variant in the phylum |
| --- | --- | --- | --- | --- | --- | --- |
| 338R-7A16T | GCT GCC **A**CC CGT AGG **T**GT | RDP | *Verrucomicrobia* | 2891 | 2955 | 97.8 |
|  |  |  | *Chloroflexi* | 537 | 2908 | 18.5 |
|  |  |  | BRC1 | 43 | 58 | 74.1 |
|  |  |  | OP10 | 53 | 181 | 29.3 |
|  |  |  | OP11 | 36 | 113 | 31.9 |
|  |  | GOS | *Verrucomicrobia* | 24 | 28 | 85.7 |
| 338R-3A7A16T | GC**A** GCC **A**CC CGT AGG **T**GT | RDP | *Planctomycetes* | 1533 | 2999 | 51.1 |
|  |  |  | OD1 | 45 | 223 | 20.2 |
|  |  | GOS | *Planctomycetes* | 5 | 10 | 50.0 |
|  |  |  | OD1 | 9 | 21 | 42.9 |
| 338R-8T15A | GCT GCC T**T**C CGT AG**A** AGT | RDP | *Proteobacteria* | 272 | 125299 | 0.2 |
|  |  |  | *Firmicutes* | 157 | 133380 | 0.1 |
|  |  |  | *Cyanobacteria* | 93 | 6870 | 1.4 |
|  |  |  | *Chloroflexi* | 54 | 2908 | 1.9 |
|  |  |  | OP11 | 11 | 113 | 9.7 |
|  |  |  | *Bacteroidetes* | 225 | 45423 | 0.5 |
|  |  |  | Unclassified Bacteria | 37 | 3211 | 1.2 |
|  |  | GOS | *Bacteroidetes* | 42 | 372 | 11.3 |
|  |  |  | *Deferribacteres* | 6 | 84 | 7.1 |
|  |  |  | *Proteobacteria* | 88 | 2756 | 3.2 |
| 338R-7C16G | GCT GCC **C**CC CGT AGG **G**GT | RDP | *Aquificae* | 663 | 700 | 94.7 |
|  |  | BisonMetagenome | *Aquificae* | 19 | 20 | 95.0 |
| 338R-16G | GCT GCC TCC CGT AGG **G**GT | RDP | *Actinobacteria* | 143 | 83391 | 0.2 |
|  |  |  | *Bacteroidetes* | 67 | 45423 | 0.1 |
|  |  |  | *Firmicutes* | 198 | 133380 | 0.1 |
|  |  |  | *Proteobacteria* | 147 | 125229 | 0.1 |
| 338R-18C | GCT GCC TCC CGT AGG AG**C** | RDP | *Proteobacteria* | 113 | 125229 | 0.1 |
|  |  |  | *Firmicutes* | 145 | 133380 | 0.1 |
|  |  |  | *Bacteroidetes* | 46 | 45423 | 0.1 |
|  |  |  | *Actinobacteria* | 121 | 83391 | 0.1 |
| 338R-7C8T15A16G | GCT GCC **CT**C CGT AG**A** **G**GT | RDP | *Cyanobacteria* | 393 | 6870 | 5.7 |
| 338R-15A | GCT GCC TCC CGT AG**A** AGT | RDP | *Bacteroidetes* | 69 | 45423 | 0.2 |
|  |  |  | *Firmicutes* | 66 | 133380 | 0.0 |
|  |  |  | *Proteobacteria* | 75 | 125229 | 0.1 |
|  |  |  | *Spirochaetes* | 21 | 2636 | 0.8 |
| 338R-15A16G17T18C | GCT GCC TCC CGT AG**A** **GTC** | RDP | *Actinobacteria* | 61 | 83391 | 0.1 |
|  |  |  | *Firmicutes* | 42 | 133380 | 0.0 |
|  |  |  | *Proteobacteria* | 77 | 125299 | 0.1 |
|  |  | GOS | *Bacteroidetes* | 17 | 372 | 4.6 |
|  |  |  | *Cyanobacteria* | 29 | 394 | 7.4 |

**Table S4. Major sequence variants at the 519F binding site.** In the “Name of the sequence variant” column, the number and capital letter after the first hyphen denote the position and nucleotide that are accordant with the degeneracy of the standard primer, while the number and capital letter following the second hyphen denote the position and nucleotide that are different from the standard primer. In the second column, nucleotides different from the standard primer appear in bold.

| Name of the sequence variant | Primer sequence  5’-CAG C**M**G CCG CGG TAA TAC-3’ | Dataset | Phylum | Number of the sequence variant | Number of sequences in the phylum | Percentage of the sequence variant in the phylum |
| --- | --- | --- | --- | --- | --- | --- |
| 519F-5A-16A | CAG CAG CCG CGG TAA **A**AC | RDP | *Chloroflexi* | 913 | 2912 | 31.4 |
|  |  |  | *Firmicutes* | 7307 | 133356 | 5.5 |
|  |  |  | *Synergistetes* | 30 | 290 | 10.3 |
|  |  |  | Unclassified Bacteria | 390 | 3215 | 12.1 |
|  |  | HumanGut | *Firmicutes* | 33 | 191 | 17.3 |
| 519F-5A-16G | CAG CAG CCG CGG TAA **G**AC | RDP | *Acidobacteria* | 195 | 4811 | 4.1 |
|  |  |  | *Actinobacteria* | 1230 | 83371 | 1.5 |
|  |  |  | *Chloroflexi* | 468 | 2912 | 16.1 |
|  |  |  | *Cyanobacteria* | 1391 | 6781 | 20.5 |
|  |  |  | *Gemmatimonadetes* | 144 | 798 | 18.0 |
|  |  |  | *Nitrospirae* | 490 | 768 | 63.8 |
|  |  |  | *Planctomycetes* | 1367 | 3001 | 45.6 |
|  |  |  | *Proteobacteria* | 1166 | 125296 | 0.9 |
|  |  |  | *Thermotogae* | 201 | 370 | 54.3 |
|  |  |  | BRC1 | 10 | 58 | 17.2 |
|  |  |  | OP1 | 28 | 28 | 100.0 |
|  |  |  | OP10 | 50 | 181 | 27.6 |
|  |  |  | OP8 | 18 | 138 | 13.0 |
|  |  |  | TG-1 | 21 | 168 | 12.5 |
|  |  |  | Unclassified Bacteria | 355 | 3215 | 11.0 |
|  |  | AcidMine | *Nitrospirae* | 51 | 57 | 89.5 |
|  |  | GOS | *Planctomycetes* | 6 | 10 | 60.0 |
|  |  | BisonMetagenome | *Chloroflexi* | 8 | 10 | 80.0 |
|  |  |  | *Cyanobacteria* | 16 | 18 | 88.9 |
| 519F-5A-16C | CAG CAG CCG CGG TAA **C**AC | RDP | *Actinobacteria* | 1087 | 83371 | 1.3 |
|  |  |  | *Chloroflexi* | 297 | 2912 | 10.2 |
|  |  |  | *Firmicutes* | 454 | 133356 | 0.3 |
|  |  |  | *Lentisphaerae* | 29 | 590 | 4.9 |
|  |  |  | *Planctomycetes* | 294 | 3001 | 9.8 |
|  |  |  | *Spirochaetes* | 1618 | 2637 | 61.4 |
|  |  | HOT | *Actinobacteria* | 4 | 17 | 23.5 |
| 519F-8G10T11A12A18G | CAG CCG CGG TAA TAC TAG | RDP | *Deferribacteres* | 115 | 732 | 15.7 |
| 519F-5A-7G8A | CAG CAG **GA**G CGG TAA TAC | RDP | OD1 | 22 | 223 | 9.9 |
|  |  | AntarcticAquatic | OD1 | 11 | 13 | 84.6 |
|  |  | GOS | OD1 | 6 | 9 | 66.7 |

**Table S5. Major sequence variants at the 907R binding site.** In the “Name of the sequence variant” column, the number and capital letter after the first hyphen denote the position and nucleotide that are accordant with the degeneracy of the standard primer, while the number and capital letter following the second hyphen denote the position and nucleotide that are different from the standard primer. In the second column, nucleotides different from the standard primer appear in bold.

| Name of the sequence variant | Primer sequence  5’-CCG TCA ATT C**M**T TTG AGT TT-3’ | Dataset | Phylum | Number of the sequence variant | Number of sequences in the phylum | Percentage of the sequence variant in the phylum |
| --- | --- | --- | --- | --- | --- | --- |
| 907R-11C-15A16T | CCG TCA ATT CCT TT**A** **T**GT TT | RDP | TM7 | 405 | 438 | 92.5 |
