# Supplementary material for: Coverage evaluation of universal bacterial primers using the metagenomic datasets
Source: BMC Microbiol. 2012 May 3;12:66. doi: 10.1186/1471-2180-12-66 (PMC3445835; doi:10.1186/1471-2180-12-66)
Supplement: Additional file 3 — Table S1; Table S2; Table S3; Table S4; Table S5. Primer binding-site sequence variants. Frequently observed sequence variants at different primer binding sites are listed in different tables: Table S1 Primer 27F; Table S2 Primer 338F; Table S3 Primer 338R; Table S4 Primer 519F; and Table S5 Primer 907R. [file 1471-2180-12-66-S3.doc]

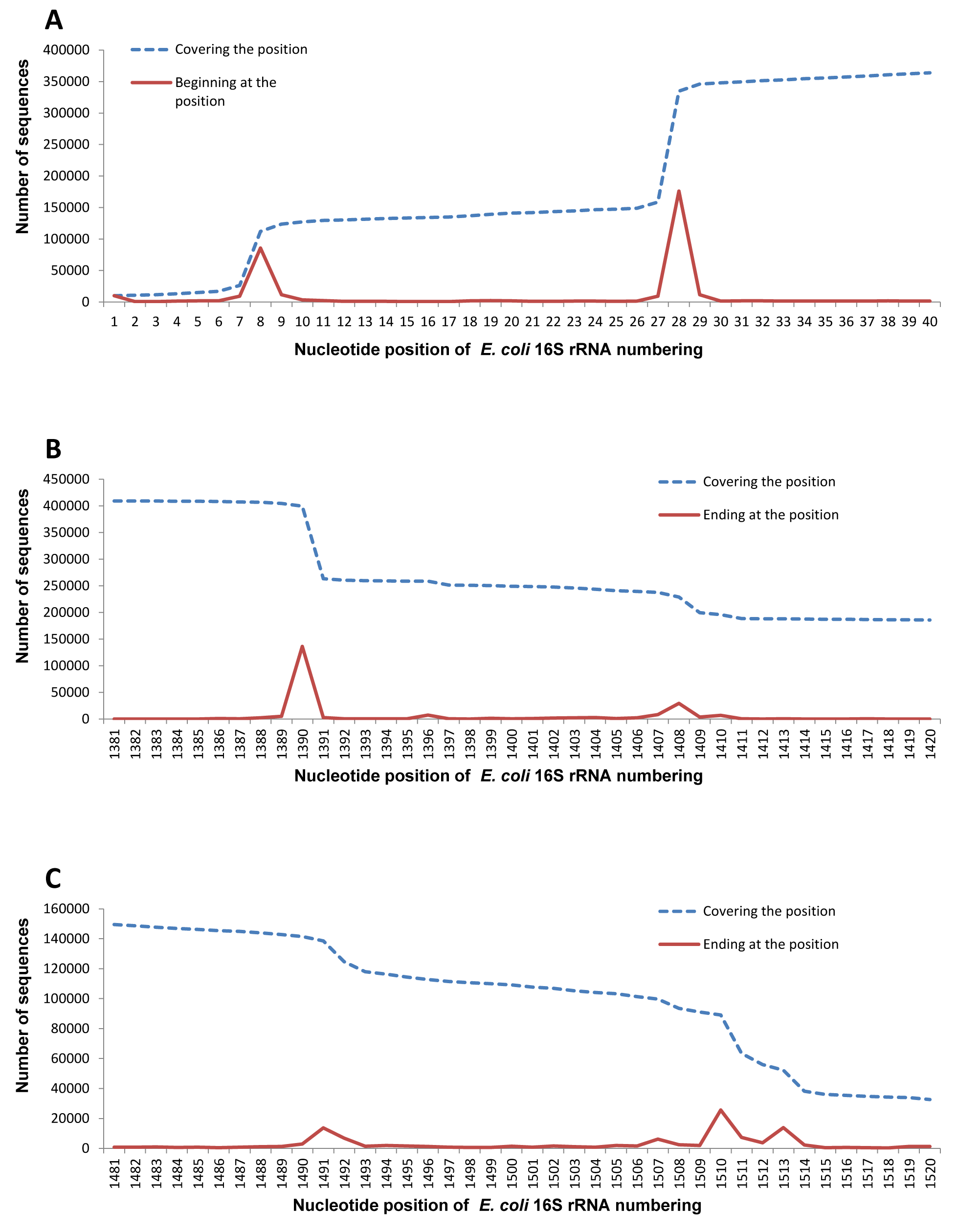


**Figure S2. Elimination of primer contamination**

The graph shows the beginning and ending positions of bacterial 16S rRNA sequences from the RDP dataset according to *E. coli* numbering. The red line shows the number of sequences beginning or ending at a given position and the blue line shows the total number of sequences that cover the position. **A** Positions 1 to 40, which include the 27F primer-binding site; **B** Positions 1381 to 1420, which include the 1390R primer-binding site; and **C** Positions 1481 to 1520, which include the 1492R primer-binding site.
