# Supplementary material for: Coverage evaluation of universal bacterial primers using the metagenomic datasets
Source: BMC Microbiol. 2012 May 3;12:66. doi: 10.1186/1471-2180-12-66 (PMC3445835; doi:10.1186/1471-2180-12-66)

**Figure S3. Non-coverage rates at the phylum level**

For each primer, phyla with non-coverage rates in at least 2 datasets are shown.

**A** primer 27F; **B** primer 338F; **C** primer 338R; **D** primer 519F; **E** primer 519R; **F** primer 907R; **G** primer 1390R; **H** primer 1492R. Abbreviations in the graphs are the same as annotated in Figure 2.


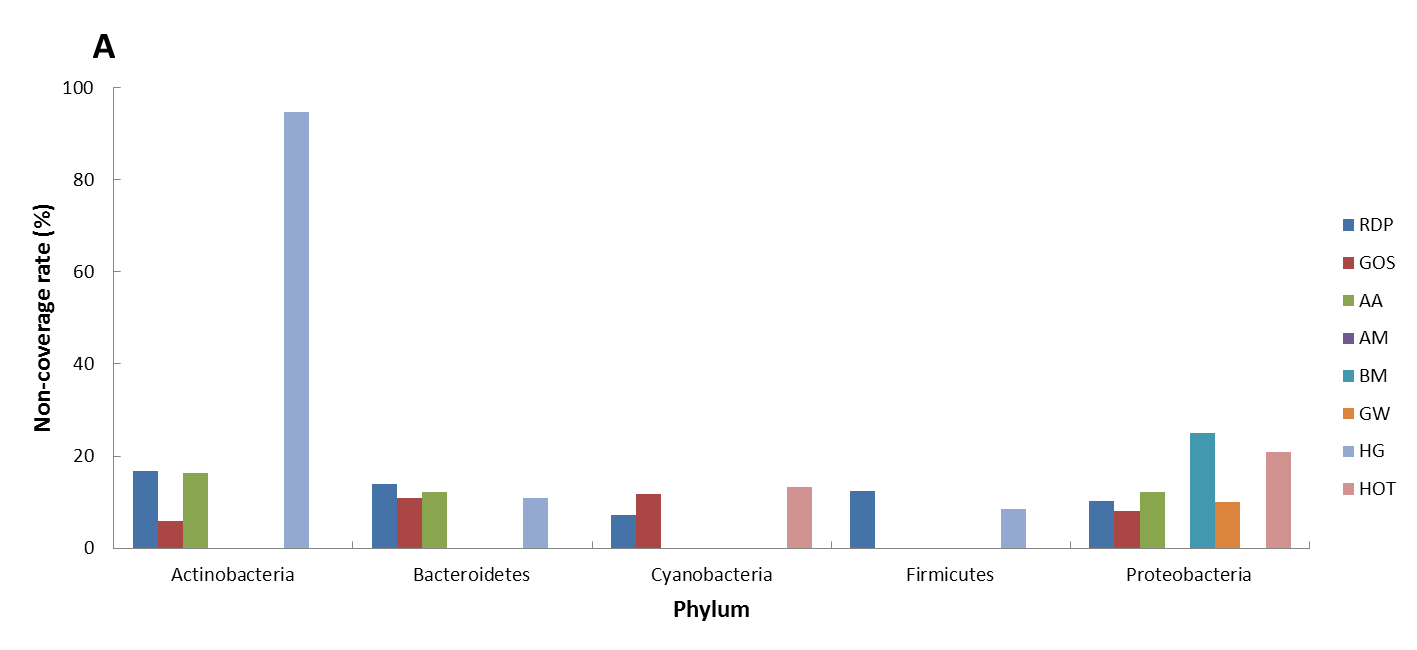


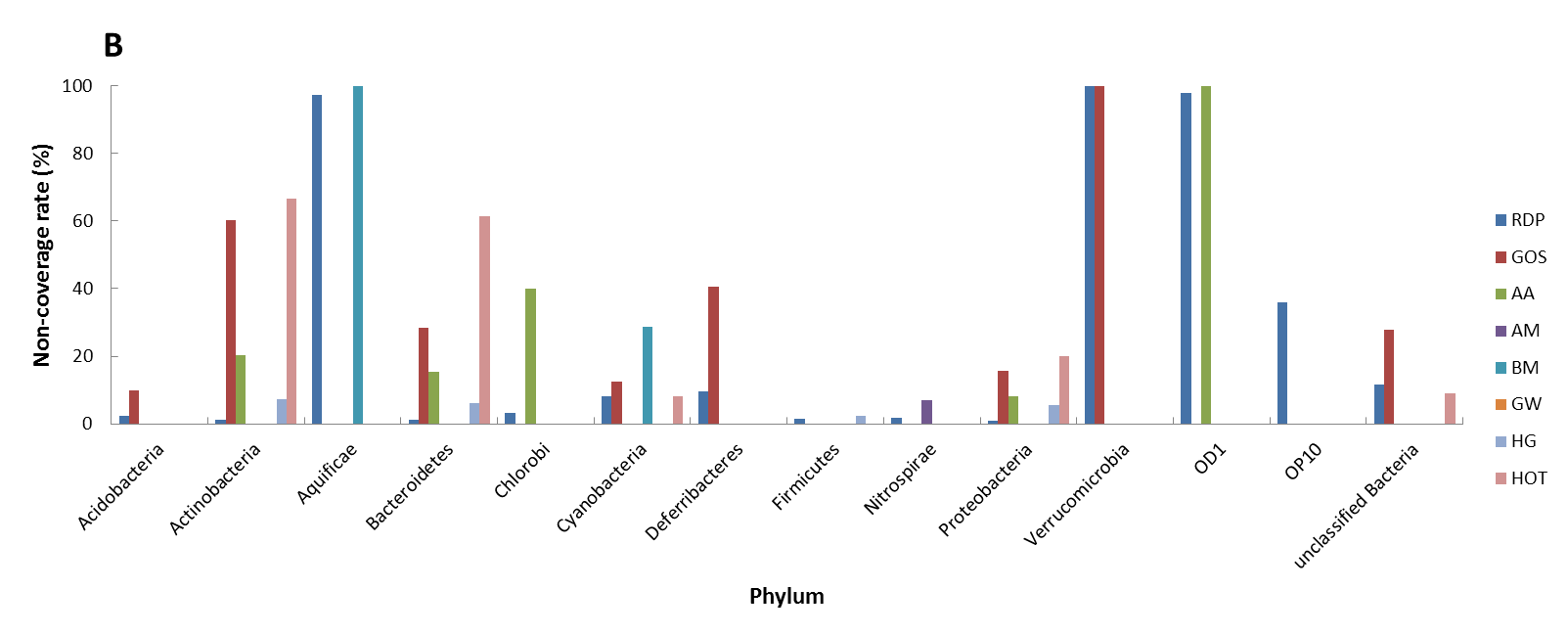


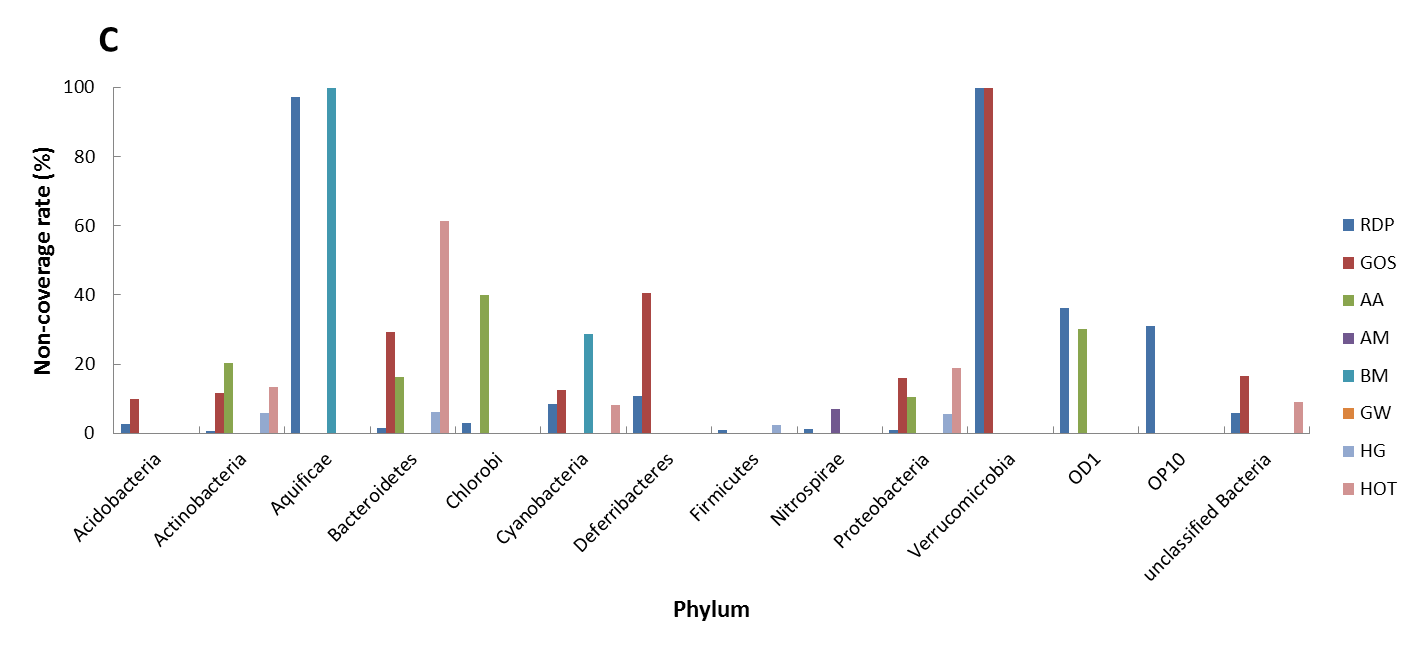


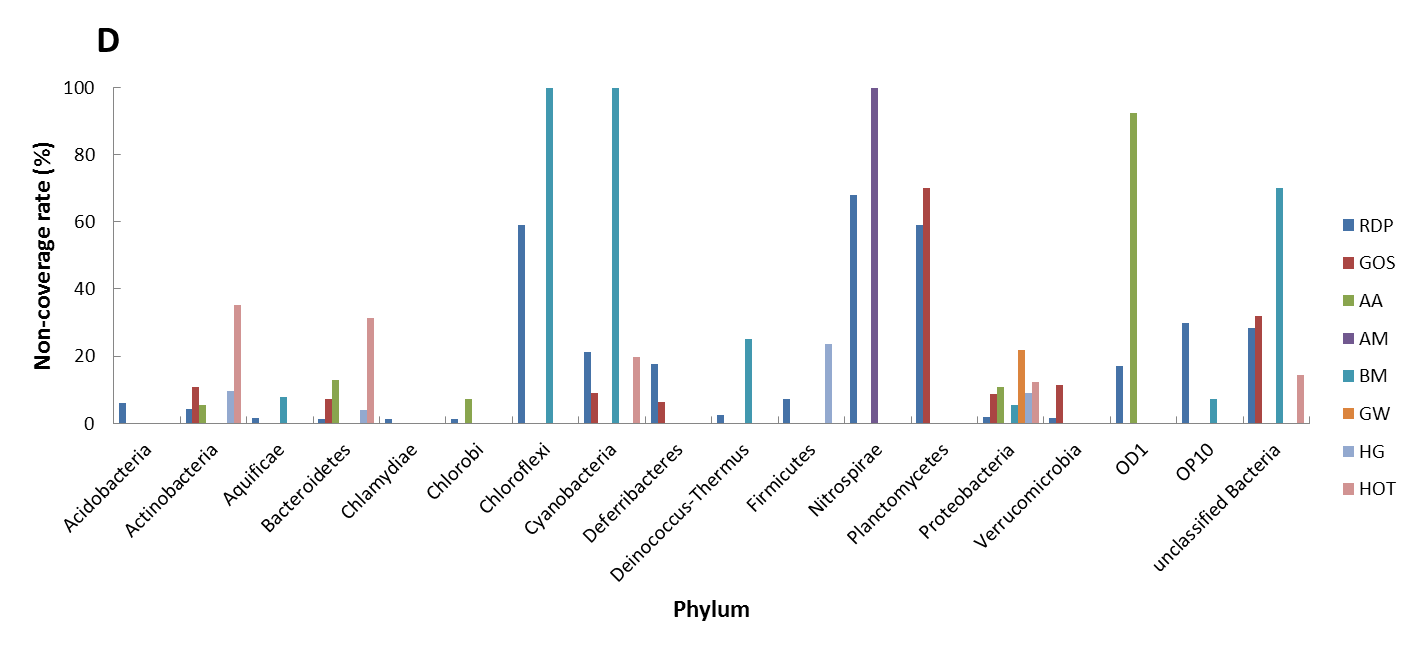


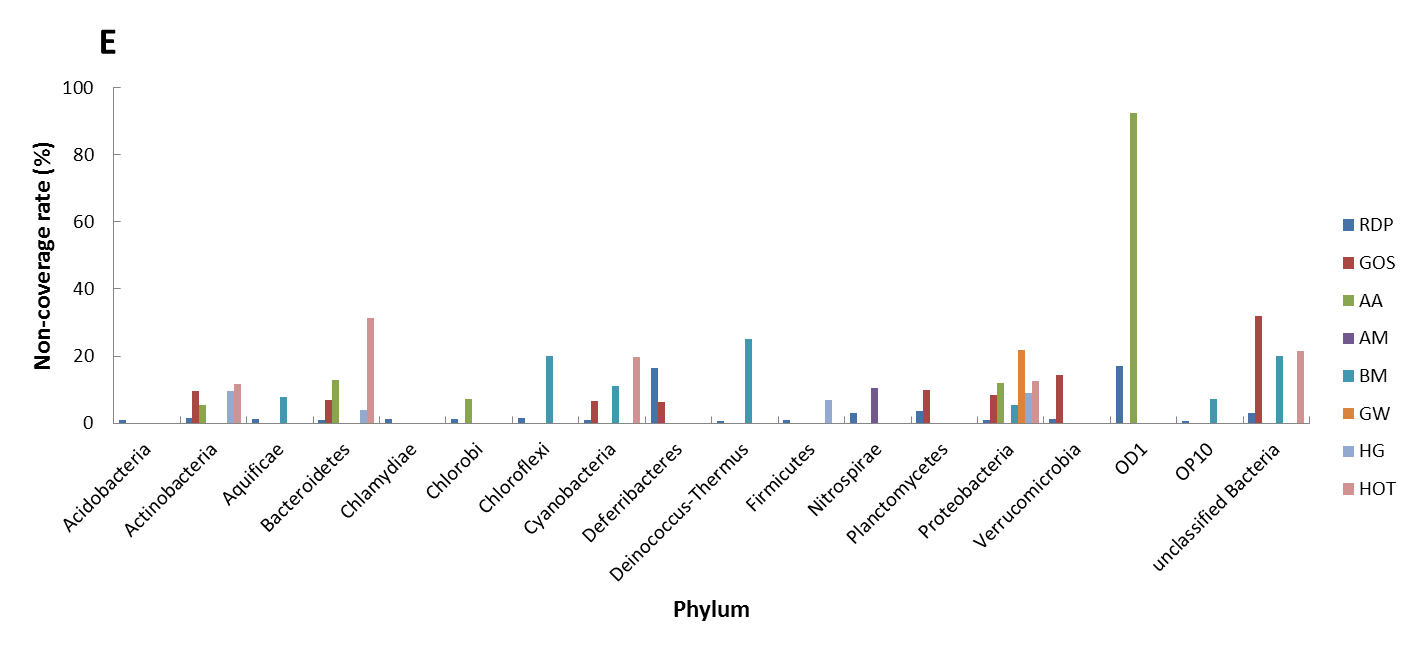


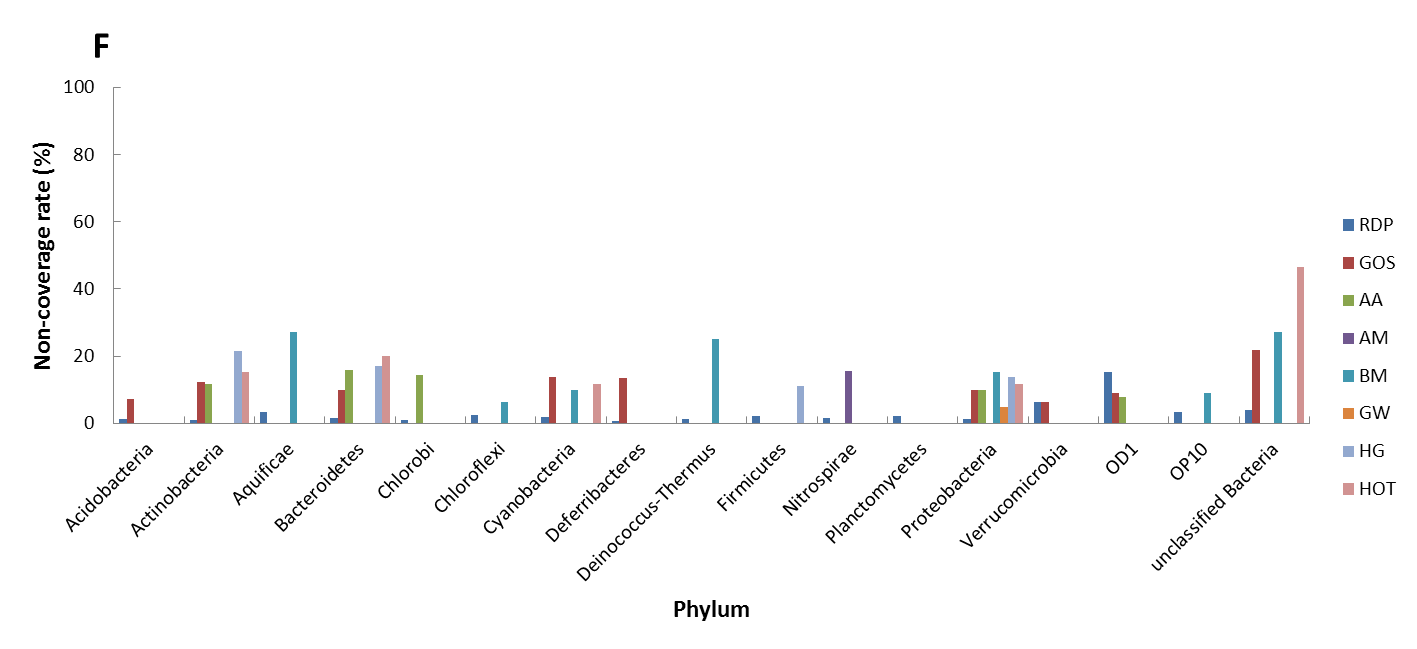


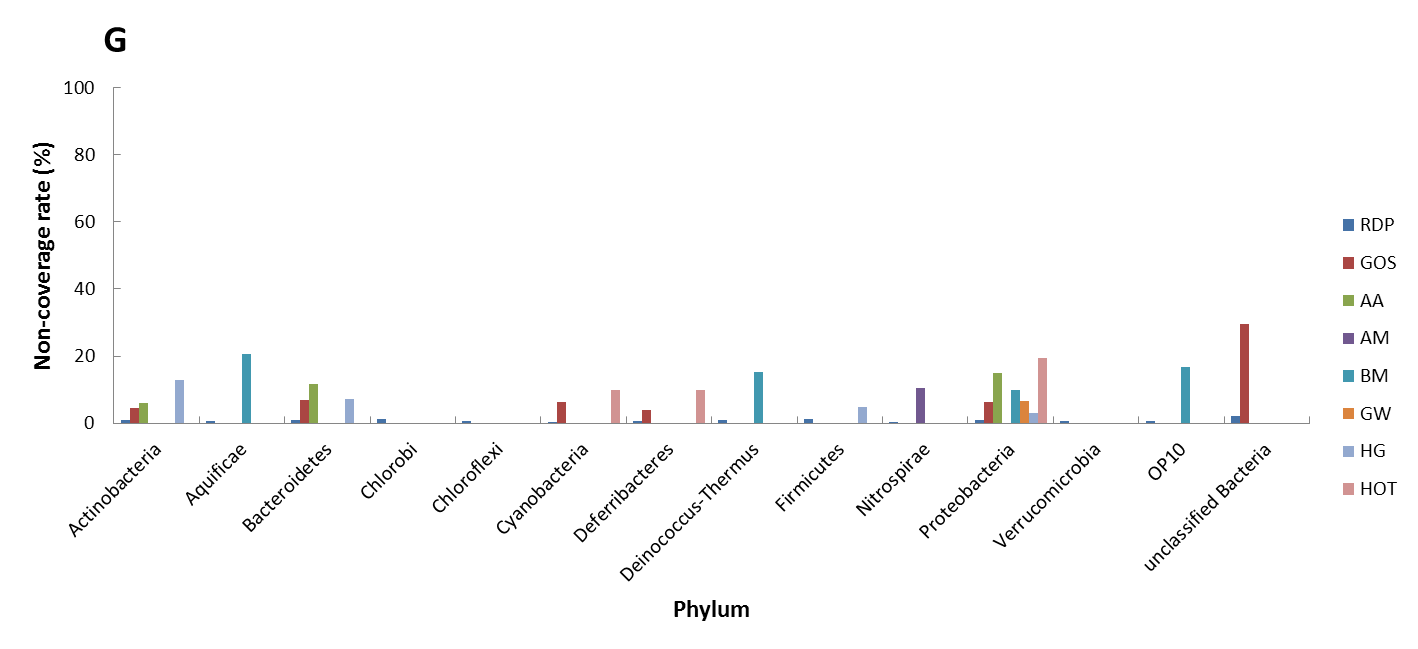


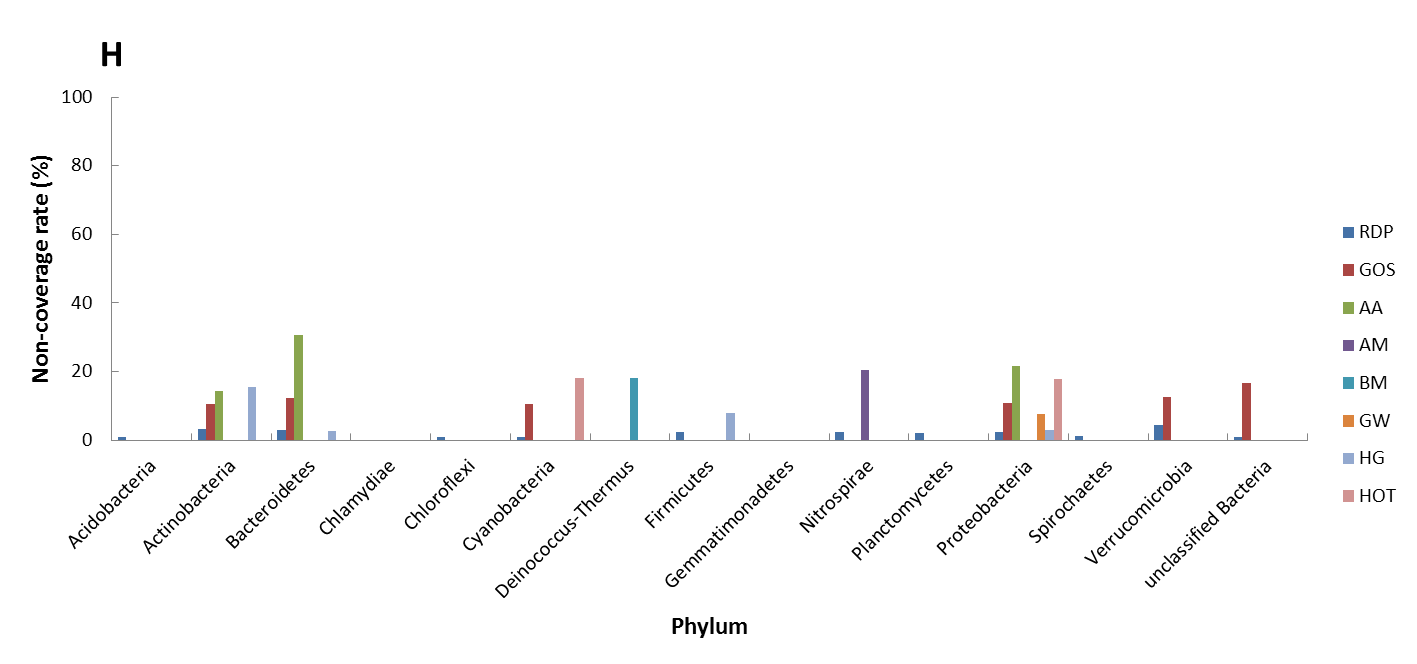

Supplement: Additional file 4 — Figure S3. Elimination of primer contamination. The figure shows the elimination of sequences that are thought to lack correct primer trimming in the RDP dataset. [file 1471-2180-12-66-S4.doc]
